# Do we need more tools or better tools to assess congestion?

**DOI:** 10.1002/ehf2.14969

**Published:** 2024-07-09

**Authors:** Jeroen Dauw, Mateusz Sokolski

**Affiliations:** ^1^ Cardiovascular Center Aalst, OLV Hospital Aalst Belgium; ^2^ Faculty of Medicine, Institute of Heart Diseases Wroclaw Medical University Wrocław Poland; ^3^ Centre for Heart Diseases, University Hospital Wrocław Poland

Assessing congestion is an essential part of taking care of heart failure patients. Congestion causes debilitating symptoms and increases the risk of short‐term clinical deterioration.^1^ It is a key pathophysiological condition, determining the need for hospitalization, but also strongly associated with prognosis, especially when presented at discharge.^2^ Research to date has demonstrated the safety and effectiveness of congestion‐targeted treatment in heart failure.^3^, ^4^ Therefore, the treating physician needs to question the presence or absence of congestion at every patient contact. Although a clinical exam is the most commonly used method to determine congestion, signs and symptoms lack sufficient sensitivity and specificity to reliably diagnose and quantify congestion. Various methods of congestion detection and quantitative assessment come to our aid, most often non‐invasive but also invasive, using implantable devices in the pulmonary artery.

A chest X‐ray is readily available and often performed to detect pulmonary congestion, but it is neither sensitive nor specific enough to be a reliable diagnostic method.^5^ Echocardiography can provide estimates of filling pressures and is an essential diagnostic tool to phenotype heart failure. However, it is also a time‐consuming exam with variable availability across Europe. Therefore, it is often logistically impossible to repeatedly perform echocardiography when treating congestion. In addition, interpretation can sometimes be difficult, and no single parameter reliably detects congestion.^6^ Thus, echocardiography is not an ideal method for the follow‐up of congestion. In recent years, different newer tools and techniques have been introduced to improve diagnostic accuracy. With ultrasound, organ congestion can be directly assessed and quantified.^2^ This includes an ultrasound assessment of the lung, renal venous flow and portal vein flow. In addition, a venous congestion scoring system called ‘VExUS’ has recently been proposed.^7^ The use of these ultrasound techniques is much less complex than echocardiography but requires the availability of a suitable ultrasound machine and minimal training of the operator. Therefore, the implementation of these ultrasound techniques seems limited to in‐hospital care and specialized outpatient clinics. In addition, the role of ultrasound methods in routine practice still needs to be determined and warrants further investigation.

Implantable haemodynamic monitors are another more invasive tool aimed at improving the detection and monitoring of congestion. A small sensor is implanted in a pulmonary artery side branch and allows for real‐time measurement of pulmonary artery pressures. Patients are asked to perform daily measurements at home, which are sent to a monitoring unit. A rise in pressure indicates congestion and occurs days before clinical signs and symptoms develop.^8^ As such, clinicians can intervene earlier and avoid overt decompensation. These haemodynamic monitors have been available for more than a decade since the pivotal CHAMPION trial was published in 2011.^9^ In patients with New York Heart Association (NYHA) class III symptoms and a previous heart failure hospitalization, the use of haemodynamic‐guided therapy was associated with a spectacular reduction of around 40% in urgent heart failure visits and hospitalizations in two large trials.^9^, ^10^ Real‐world European data confirmed these trial findings.^11^, ^12^, ^13^ However, the device comes with high costs, requires an invasive procedure and is not yet reimbursed in a lot of European countries. Furthermore, there seems to be no benefit for patients in a less severe disease state.^14^ At this stage, it must be emphasized that pressure‐based measurements do not always reflect the intravascular condition, and pressure does not equal volume.^15^ It is possible that integrating many standard parameters, including clinical assessment, laboratory tests and data from cardiac implantable electronic devices when available, would be more effective in hospital and post‐hospital settings.^16^, ^17^

Remote dielectric sensing, or ReDS, is a novel, non‐invasive tool to assess pulmonary congestion. It uses a radiofrequency‐based sensor (comparable to radars) to measure pulmonary water content, expressed as a percentage. A radiofrequency wave is generated at the patient's back and is transmitted through the thorax and lungs until it reaches the detector on the opposite side. By analysing the travel time and power of the detected waves, lung fluid content can be reliably calculated. A single measurement takes around 45 s and can be performed on top of clothes. A non‐congested lung contains between 25% and 35% water. A higher percentage indicates congestion, while a lower percentage indicates dehydration. ReDS has been proven to be accurate, and its use might reduce 30 day heart failure events, as shown in a small proof‐of‐concept study.^18^

Izumida *et al*. studied the prognostic value of a ReDS in 133 patients admitted with heart failure at a single tertiary care centre in Japan.^19^ The authors created a ‘ReDS ratio’, comparing the ReDS absolute value on admission with the absolute value at discharge, with lower numbers (<100%) indicating a decrease and higher numbers (>100%) indicating an increase in lung fluid. Unfortunately, the authors chose to also express the ReDS ratio as a percentage, which inevitably leads to confusion. Of note, the majority of patients had no pulmonary congestion. The median ReDS value on admission was 29%, with a 25th–75th percentile range of 25%–33%. This means that at least half of patients had a normal ReDS value (25%–35%). As expected, a ReDS ratio > 100%, indicating an increase in lung fluid, was associated with a worse prognosis. However, the added value of this ratio is very questionable. Possibly, only using absolute differences might also be sufficient and, above all, easier, but this was not studied by the authors. Moreover, the advantage of ReDS is precisely that it is able to provide absolute numbers of lung fluid. Therefore, the ReDS ratio just seems to complicate the interpretation. Apart from this fact, the study demonstrates the weakness of conventional parameters to follow up on pulmonary congestion. Patients with a ReDS ratio > 100%, surprisingly, had a significant decrease in body weight and/or natriuretic peptide during the hospital course. In addition, in patients with a ReDS ratio > 100%, there was no significant increase in the use of loop diuretics, sodium–glucose cotransporter 2 inhibitors or tolvaptan at discharge. In general, the percentage of loop diuretics and sodium–glucose cotransporter 2 inhibitors administered was quite low in the entire population, which may result in suboptimal in‐hospital treatment effects but also in the long‐term prognosis. Currently, heart failure therapy allows for a very effective treatment, including a reduction in congestion. Therefore, the lower use of heart failure drugs could also explain why these patients had no improvement in the ReDS measurements. Heart failure treatment should be implemented as early as possible, can be started already in the acute phase and aim to rapidly achieve maximally tolerated doses. Adequately dosed heart failure drugs reduce symptoms, improve quality of life and improve the prognosis significantly. High‐intensity care after an acute heart failure event is even safer and more beneficial in an elderly population.^20^ The median age of the studied cohort was 78 years old, which is considerably older than other studies in this field. Probably, older patients benefit at least as much as others from improved congestion detection and treatment as with ReDS, but this requires further studies. An analysis of kidney function during hospitalization could be a valuable element, but unfortunately, these data were not collected. Renal failure itself may influence the prognosis, and the guidance of heart failure by remote monitoring may be more efficient in patients without chronic kidney disease.^21^, ^22^

Concluding, ReDS is an interesting tool as it is accurate, reproducible and provides actionable information, warranting further studies. A better assessment of congestion with new technologies and integrating these measures with standard clinical data may be the way to go to improve heart failure management. Nevertheless, we do not just need more tools; we also need better tools that improve the outcomes of our patients.

## Conflict of interest statement

The authors declare no conflicts of interest.
